# The Patient With Multiple Alloantibodies: A Systematic Strategy for Antibody Identification and Transfusion Management

**DOI:** 10.1155/crii/4700436

**Published:** 2026-09-02

**Authors:** Ahmed Alharbi, Haya Wasel, Eman Almutairi, Nourah AlHarethi, Abdulmohsen Alotaibi, Jalal Hassan, Hiyam Almutairi, Rayyan Alotaibi, Maram Alonayzan, Sarah Abdullah Alobaidi, Sarah Al Otaibi, Majd Al Anazi, Hajer Aziz, Rawan Alfraidi, Sanad Al Harthi, Saleh Almutairi, Ahmed Shareefi, Bandar Al Qahtani, Atheer Aloqayli

**Affiliations:** ^1^ Department of Pathology and Laboratory Medicine, King Abdullah Specialist Children Hospital – KASCH, King Abdulaziz Medical City-CR, Ministry of National Guard, Riyadh, Saudi Arabia, mngha.med.sa

**Keywords:** anti-E, anti-N, anti-P1, anti-s, antibody identification, cold-reactive antibodies, multiple alloantibodies, transfusion management

## Abstract

**Background:**

For patients with multiple alloantibodies against red blood cells (RBCs), the challenges posed by transfusion practice are immense due to complex serological reactions that make finding matching units difficult. Identifying underlying antibodies is necessary to prevent hemolytic transfusion reactions (HTRs).

**Objective:**

To outline a systematized immunohematologic strategy for the detection and management of several alloantibodies in a surgical patient.

**Methods:**

A 64‐year‐old O‐positive female was scheduled for spinal surgery and had a positive pre‐transfusion antibody screen. Automated and manual techniques, including multi‐cell panels, enzyme‐treated cells, thermal amplitude testing, extended phenotyping, DAT, and acid elution, were used in antibody investigation.

**Results:**

The alloantibodies anti‐s and anti‐E were proven to be warm‐reactive IgG. The cold‐reactive IgM alloantibodies anti‐P1 and anti‐N were similarly shown to exhibit characteristic temperature reactivity. Anti‐Fyb was suspected, but not excluded, for a lack of homozygous rule‐in cells. Eluates showed an anti‐s reaction with transfused RBCs, consistent with recent exposure. Antigen‐negative, phenotype‐compatible RBCs were selected that only reacted in the antiglobulin stage, although spin incompatibilities were immediate.

**Conclusion:**

This case highlights the significance of systematic approaches to antibody rule‐in/rule‐out, the thermal‐phase test, and phenotype‐directed transfusion in patients with multiple alloantibodies. A clear distinction must be made between clinically significant IgG antibodies and cold‐reactive IgM antibodies to ensure safe, timely transfusion management.

## 1. Introduction

Although the presence of a single red blood cell (RBC) alloantibody is relatively common, the accumulation of multiple alloantibodies is increasingly recognized among patients who have received multiple transfusions, such as those with thalassemia, sickle cell disease, myelodysplastic syndromes, or post‐transplant patients [1, 2]. The rate and specificity of alloantibody production are determined by various factors, which include patient antigenic exposure, genetic predisposition, and antigenic differences between patients and recipients [3].

The presence of multiple alloantibodies complicates serological analysis and makes finding a compatible RBC unit for transfusion more difficult. The higher the number of alloantibodies, the higher the risk of incompatible results of crossmatches, transfusion reactions, and hemolytic transfusion reactions (HTRs) [4]. In some cases, the urgency of transfusion may necessitate the prudent use of units with insufficient match, but this should be carefully balanced against the risk of alloimmunization and potential adverse reactions [5].

Serological techniques that assess reactivity at different stages of the test can be used to identify alloantibodies. The test stages are as follows: Immediate spin at RT; incubation at 37°C; and the antiglobulin test (AHG) at the AHG Phase [6]. At 4°C, it may be necessary to test to determine the optimal reactivity of certain cold‐reactive antibodies, for example, anti‐P1 [7]. Thermal amplitude of each antibody, as well as reaction strength, should be well defined, particularly in the presence of multiple alloantibodies and their reaction, which may produce overlapping or masking effects.

Comprehensive testing strategies, including extended phenotyping, enzyme‐treated panels, thermal phase manipulation, and automated and manual methods, are essential for identifying the specificity of each antibody. This will ensure the availability of phenotypically matched, antigen‐negative blood units and will ultimately improve patient safety and clinical outcomes for multi‐alloimmunized patients [8, 9].

## 2. Case Presentation

The patient was a 64‐year‐old woman with hypothyroidism and spinal surgery that was conducted 20 years ago. According to the blood bank’s laboratory information system, the patient was group O positive and had a previous, nonspecific antibody detected during a prior admission. At that time, the direct antiglobulin test (DAT) was negative, and we were able to perform RBC phenotyping.

A total of two units of crossmatch‐compatible RBCs were administered to the patient 10 days before the current hospital course. Since there had been no indication of a specific alloantibody up to that point, the blood had not been phenotyped.

During the present admission, a routine type and screen demonstrated that she was O‐positive and had a strongly positive antibody screen; accordingly, the process for antibody identification began. The preliminary process using the standard 14‐cell identification panel indicated the presence of an anti‐s. Nevertheless, the rule‐out also indicated that other significant alloantibodies may be present and could not be eliminated even in the simplest reactions, as suggested by the results.

Thus, the comprehensive antibody identification panel was performed using panel cells and enzyme‐treated cells, and testing was conducted across various thermal phases. DAT was weakly reactive with anti‐IgG and nonreactive with anti‐C3d. The acid elution technique confirmed the anti‐s coating present on the patient’s RBCs, establishing the presence of allo‐anti‐s on the RBCs, likely from transfused units rather than auto‐production. This correlated with the patient’s previous antigen‐negative status and transfusion history.

By thermal amplitude testing, marked reactivity at 4°C and fading reactions at 37°C and during the antiglobulin stage were observed, typical of cold‐reacting IgM antibodies. Anti‐P1 was thus distinguishable by these reactions to these different temperatures. Further studies using selected cells indicated the existence of anti‐N. However, the presence of anti‐Fyb could not be excluded because homozygous cells were not available for the studies.

Further confirmation studies using the tube technique and ficin‐treated cells (Immucor Panocell 10 cells) confirmed the increased reactions and the presence of anti‐s, anti‐P1, anti‐N, and possibly anti‐Fyb. The anti‐P1 antibody exhibited typical cold reactions, with maximal responses at 4°C, whereas responses were lost at 37°C and during the AHG phase, confirming its IgM class. However, the possibility of additional cold agglutinins could not be excluded.

Although antibody testing was not required immediately, sufficient time was allowed for serologic analysis. Postoperatively, the patient developed a decrease in hemoglobin levels; therefore, transfusion was needed. RBCs phenotypically identical units, omitting antigens s, N, E, and Fyb, were chosen. The chosen units showed chilled incompatibility in the immediate spin phase due to cold antibody interference; however, compatibility was demonstrated in the 37°C and AHG phases. Cold‐repeat antibody testing demonstrated strong cold reactivity typical of antibodies against antigen P1.

Two compatible units of phenotype‐matched RBCs were transfused uneventfully following surgery. Healthcare personnel were instructed to warm the blood, if needed, to reduce the risk of cold antibody reactions.

## 3. Methods

Pre‐transfusion whole blood specimens were obtained by venipuncture and collected in 7 mL EDTA tubes (BD Vacutainer K2E EDTA, 10.8 mg of EDTA). Pre‐transfusion specimens were centrifuged at 4800 rpm for 10 min at room temperature. After separation, the specimens were refrigerated at 2–10°C for up to 14 days.

The ABO reverse grouping, reverse typing of the RhD antigen, and phenotyping of the Rh antigen were carried out using an automated microplate Immunohematology Analyzer (Immucor NEO/Neo Iris; Immucor Inc., Norcross, GA, USA). This microprocessor‐based system is designed to automate data processing and analysis of data generated in routine in vitro immunohematology tests, such as blood group determination.

Screening of antibodies and initial antibody identification were done as per the guidelines of the manufacturer using Capture R Ready ID and Capture c Ready Screen reagents (Immucor Inc.) on the same instrument. Further antibody identification, enzyme typing, and thermal amplitude assessment were done by manual tube and gel methods (Immucor Inc.). The ficin treatment of RBCs was used as the reagent in enzyme typing, and the reactions were read as enhanced, reduced, or destroyed according to conventional definitions. The reaction strength was scored on a 0–4+ scale.

The DAT was done using monospecific anti‐IgG and anti‐C3d reagents by the manual gel technique (Bio‐Rad DC‐Screening II, DiaMed GmbH, Cressier, Switzerland). Acid elution was done on DAT‐positive samples according to standard acid elution test methods. Red cell phenotyping was also performed on the various RBCs using the manual gel technique (DiaMed GmbH).

The antibody identification was performed using defined criteria for rule‐in and rule‐out reactions, including reactivity of the serum with antigen‐positive cells but not with antigen‐negative cells; use of homozygous cells when available; correlation with the patient’s RBC phenotype; and reactivity within the phase boundaries.

In pre‐transfusion testing, a total of 7 mL EDTA blood was needed for ABO and Rh blood grouping, compatibility testing, and antibody screening. Such type and screen results are valid for only 72 h after blood collection. In patients with no recorded ABO and RhD blood types, two separate samples were obtained and analyzed. In fact, ABO and Rh blood typing and antibody screening for unexpected antibodies in patients’ red cells were performed. These patients had their ABO and RhD blood groups reconfirmed before transfusion.

Summary of identified and suspected alloantibodies.

**Table:** 

Antibody	Status	Evidence	Serologic characteristics	Clinical relevance
Anti‐E	Confirmed	Panel reactivity; enhanced with enzyme‐treated cells; reaction at 37°C and AHG phase	Warm‐reactive IgG; enzyme‐enhanced	Clinically significant; associated with hemolytic transfusion reactions (HTRs); requires antigen‐negative blood.
Anti‐s	Confirmed	Panel reactivity; confirmed by DAT (IgG+) and acid elution; reduced/destroyed by enzyme treatment	Warm‐reactive IgG; enzyme‐sensitive (destroyed in enzyme treatment)	Clinically significant; implicated in HTRs; necessitates s‐negative units
Anti‐P1	Confirmed	Strong reactivity at 4°C; non‐reactive at 37°C and AHG; thermal amplitude testing	Cold‐reactive IgM; temperature‐dependent	Usually clinically insignificant; may interfere with immediate‐spin crossmatch
Anti‐N	Confirmed	Identified using selected cells; cold‐phase reactivity pattern	Cold‐reactive IgM	Generally clinically insignificant; may cause serologic interference
Anti‐Fyb	Suspected (not excluded)	Incomplete rule‐out due to lack of homozygous cells; panel limitations	Likely IgG; dosage effect suspected	Potentially clinically significant; precautionary antigen‐negative selection recommended

**Figure 1:**
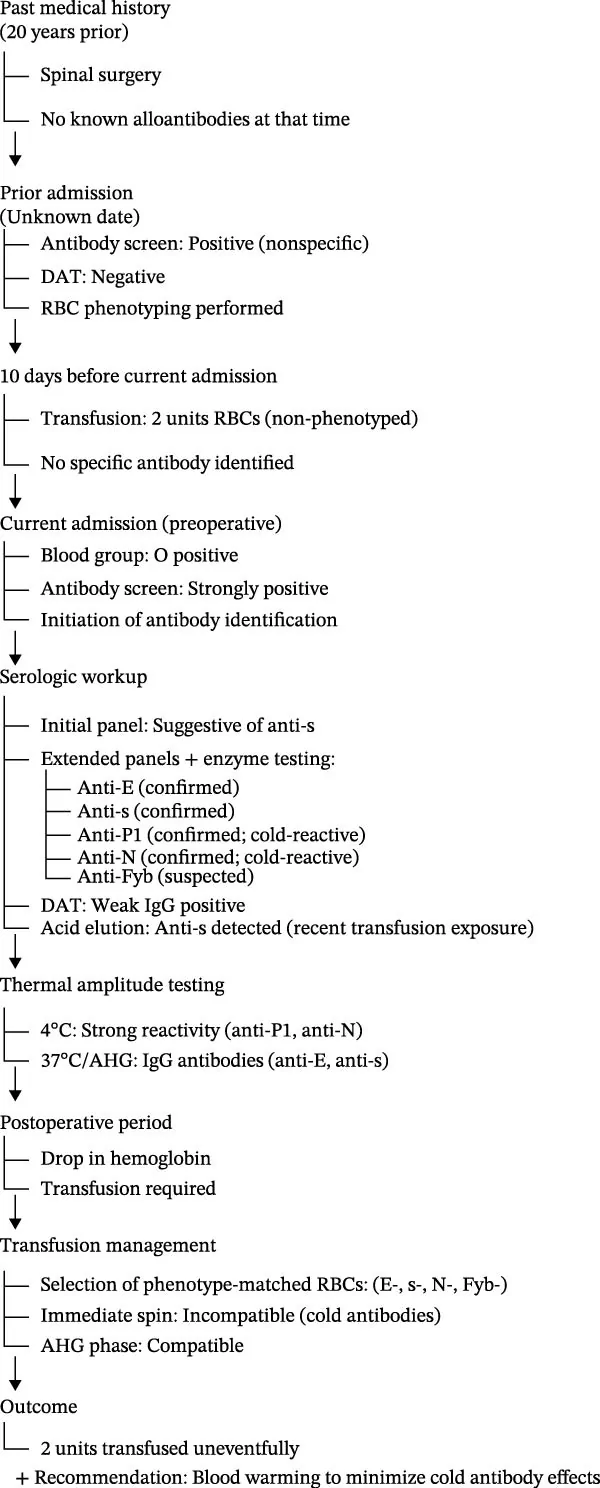
Clinical and serologic timeline of antibody development and transfusion management.

In this figure, timeline illustrating the patient’s transfusion history, antibody development, serologic investigations, and transfusion management. The sequence highlights the emergence of multiple alloantibodies following a recent transfusion, the differentiation of warm and cold antibodies through thermal and enzyme testing, and the successful selection of phenotype‐matched RBC units (Figure 1).

## 4. Results

The patent’s baseline immunoheamatologic findings, including ABO/RHD blood group, red cell phenotype and DAT are shown in Tables 1–5.

**Table 1:** ABO grouping results.

ANTI‐A	ANTI‐B	ANTI‐D	RH ctr	AC	BC
0	0	4	0	4	4

**Table 2:** Rh phenotype results.

C	C	E	e	K
4	0	0	4	0

**Table 3:** Extended phenotype results.

P1	Lea	Leb	Lua	Lub	Kpa	Kpb	Jka	Jkb	M
0	0	4	0	3	0	2	4	3	2

**Table 4:** Extended phenotype results (continued).

N	Fya	Fyb	S	s	k
0	2	0	2	0	3

**Table 5:** DAT results.

AHG IgG	C3d	Ctr
Weak positive	0	0

**Figure 2:**
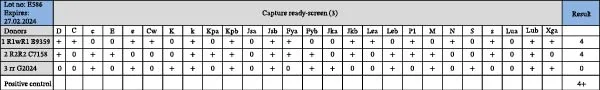
Antibody screening panel.

**Figure 3:**
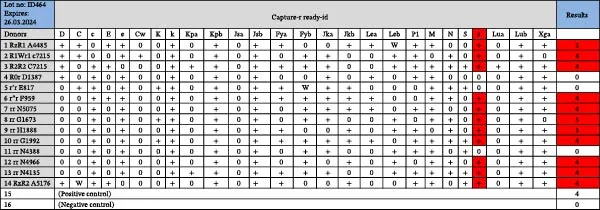
Antibody identification panel. This panel indicates the presence of anti‐s, additional clinically significant alloantibodies could not be excluded. RBC phenotyping assisted in antigen exclusion.

**Figure 4:**
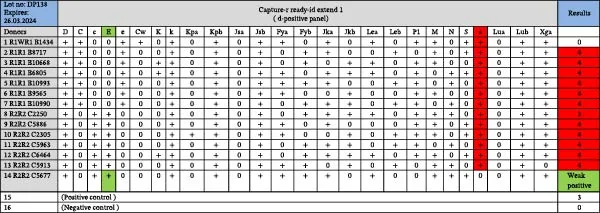
Antibody identification panel (extended). This panel confirms the presence of anti‐s, the possible presence of anti‐E (cell 14); other clinically significant alloantibodies could not be excluded.

**Figure 5:**
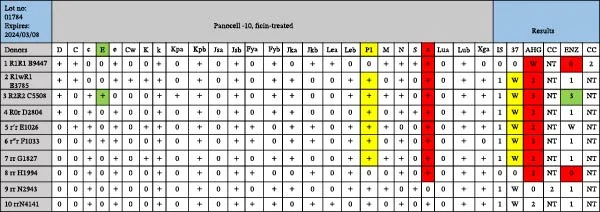
This panel indicates the presence of anti‐P1 at immediate spin phase, additionally the presence of anti‐s and more confirmation in cell 1 throughout cell 8, and the reaction was denatured following enzyme treatment (cell 1 and 8). The presence of anti‐E was confirmed due to enhanced reactivity following enzyme treatment in cell number 3. Other clinically significant alloantibodies could not be excluded.

**Figure 6:**
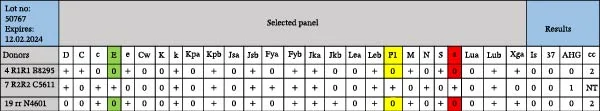
Selected panel cells used to evaluate and exclude anti‐Fyb.

**Figure 7:**
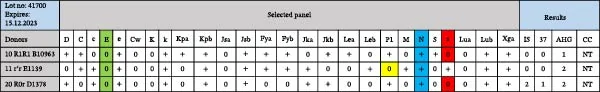
Selected panel cells demonstrating the presence of anti‐N.

**Figure 8:**
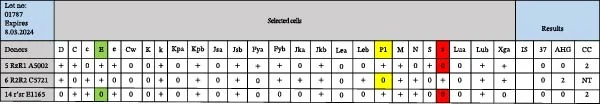
Selected panel cells used to exclude anti‐C.

The antibody investigation was performed in a stepwise manner using antibody screening, identification panels, enzyme‐treated cells, selected cells, and thermal phase testing. The antibody screening result is shown in Figure 2. The initial identification panel suggested the presence of anti‐s, while additional clinically significant antibodies could not be excluded (Figures 3 and 4). Further testing with enzyme‐treated cells confirmed anti‐E and anti‐s and demonstrated the characteristic cold‐reactive pattern of anti‐P1 (Figure 5). Selected‐cell studies were then used to evaluate and exclude anti‐Fyb, confirm anti‐N, and exclude anti‐C (Figures 6–8). The overall finding supported the selection of phenotype‐matched RBC units for transfusion.

## 5. Discussion

The difficulty in diagnosing and managing a patient who produces several RBC alloantibodies at the same time, who is shown to possess warm‐reactive IgG alloantibodies against RBCs (anti‐E and anti‐s), cold‐reactive IgM alloantibodies (anti‐P1 and anti‐N), and is presumed to possess alloantibodies anti‐Fyb, can be seen in the literature on blood transfusions. The association between cold and warm autoagglutinins has been reported [10–12].

If anti‐s antibodies were detected in the eluate despite being negative for s, this would explain the s‐negative phenotype in a patient due to possible transfusion of positive donor RBCs, as seen in positive DAT results post‐transfusion [13, 14]. Accurate interpretation of antibody specificity requires careful correlation of elution findings with transfusion history and DAT results to avoid misleading conclusions [15–17]. Although anti‐P1 and anti‐N antigens are generally not significant, reactions can affect antibody screening and crossmatching, especially at lower temperatures [18, 19]. The lack of P1 neutralization testing in this situation is a known limitation when specialized reagents are not readily available [20].

Multifactorial alloimmunization, as observed in this patient, is increasingly reported among chronically transfused individuals, especially those with underlying hematologic disorders or repeated transfusion exposure [21, 22]. The presence of multiple alloantibodies makes it difficult to identify compatible RBC units, increases transfusion turnaround time, and elevates the risk of HTRs [23].

The primary antibody screen demonstrated high serologic reactivity, necessitating further evaluation with standard 14‐cell identification panels. It has already been shown that patients with multiple alloantibodies often require repeated testing across various panel lots, using enzyme‐treated cells and under a wide range of thermal conditions to fully characterize antibody specificity [24–26]. Initial panel findings may indicate a single antibody, whereas additional or masked specificities may be detected through prolonged testing [27]

This case required enzyme treatment to distinguish the specific properties of the antibody. Both anti‐E and anti‐s are IgG antibodies reactive at 37°C and in the AHG phase, but anti‐s can be weakened or destroyed by enzyme treatment; moreover, Rh antibodies, such as anti‐E, are normally enhanced [28, 29]. The selective loss and stimulation of reactivity after enzyme treatment have been broadly reported as useful techniques in resolving overlapping antibody patterns [30]. These results support published guidelines recommending the use of complementary manual and automated techniques to assess antibody strength and temperature dependence [5, 6], see Figures 3 and 4.

The identification of anti‐P1 underscores the importance of cold‐phase testing when warm‐reactive antibodies do not fully account for serologic findings. Anti‐P1 is an IgM antibody commonly detected in individuals with the P2 phenotype and shows strong cold reactivity, diminished activity at 37°C, and AHG phase activity [20, 21]. Although anti‐P1 is rarely associated with clinically significant hemolysis, it is a well‐recognized source of serologic interference during immediate‐spin crossmatching [20], see Figure 5.

Suspected anti‐N and anti‐Fyb were investigated using selected cell panels. While anti‐N could be demonstrated, anti‐Fyb could not be definitively confirmed due to the lack of homozygous reagent cells. The need for homozygous expression to exclude dosage effects is well established, and limited access to such panels remains a challenge in many transfusion services, particularly in resource‐limited settings [22, 23], see Figures 6 and 7.

Providing phenotype‐matched blood is a cornerstone of transfusion support for patients with multiple alloantibodies. In this case, RBC units were selected as negative for all identified and suspected antigens. Weak incompatibility at the immediate‐spin phase, with compatibility at the AHG phase, has been observed in the presence of cold‐reactive antibodies. It is regarded as an acceptable practice when the clinically significant antibodies have been ruled out [20, 21]. This approach complies with AABB and international transfusion guidelines that recommend extended antigen matching beyond ABO and RhD in alloimmunized patients to reduce the risk of HTR [5, 6]

The case also supports the role of prophylactic antigen matching in the high‐risk patient population. Alloimmunization is common among patients with thalassemia, sickle cell disease, and myelodysplastic syndromes, and various studies have shown that prophylactic Rh and Kell matching can significantly reduce antibody formation and transfusion complications [1, 18].

Another issue in this case is the evanescent nature of the alloantibodies. The antibodies may decrease to undetectable levels with time and re‐emerge when exposed to the antigen. This phenomenon is well described and warrants a thorough review of the historical antibody record [25, 26]. Effective laboratory information systems and long‐term monitoring of antibodies are thus the key to safe transfusion practice [19].

Lastly, this case highlights the need for close collaboration between transfusion services and clinical teams. Postponing transfusion until compatible units are available, even in emergencies, is evidence‐based because studies have shown that when serologic compatibility is considered an important factor, there are fewer hemolytic complications [6, 27]. The logistical issues faced also underscore the role of rare donor registries and extended‐phenotype or even genotype databases, which have been found to better provide access to compatible blood for multi‐alloimmunized patients [16, 17].

A formal neutralization assay to ascertain the specificity of anti‐P1 was not done because of limitations on the availability. It was confirmed by characteristic cold‐phase serologic behavior, disappearance at 37°C, AHG, and reproducibility across panel cells. Although this method is consistent with current laboratory practice, the absence of neutralization testing limits the ability to exclude other cold‐reactive IgM antibodies [20, 21].

Although anti‐P1 was identified based on its characteristic serologic profile, namely, strong reactivity at 4°C with loss of activity at 37°C and in the AHG phase, formal neutralization testing was not performed due to limited reagent availability. This introduces residual uncertainty, as other cold‐reactive IgM antibodies with similar thermal‐amplitude patterns cannot be completely excluded. Nevertheless, the reproducibility of reactions across multiple panel cells and the consistent temperature‐dependent behavior support the presumptive identification of anti‐P1. Future investigations could strengthen antibody confirmation by using specific neutralization assays (e.g., soluble P1 substance) or adsorption techniques, as well as by accessing extended reagent panels, to exclude coexisting cold‐reactive specificities definitively. In resource‐limited settings, a minimal workflow may include (1) assessment of thermal amplitude, (2) reproducibility across multiple panel cells, (3) evaluation of auto control and DAT, and (4) testing against antigen‐negative cells when available. Referral to a reference laboratory is recommended if results remain inconclusive.

While the approach is broadly applicable, this report represents a single case and should be interpreted cautiously. In settings with limited reagents (e.g., lack of homozygous/heterozygous cells), the workflow can be adapted by emphasizing thermal amplitude assessment, reproducibility across available panel cells, and referral to reference laboratories when needed. In larger programs, incorporation of extended phenotyping or genotyping and engagement with rare donor registries can enhance antibody confirmation and support access to compatible blood units.

## 6. Limitation

This report describes a single patient and, as such, the findings may not be fully generalizable to all transfusion settings or patient populations. The comprehensive immunohematology workup performed in this case, including extended panels, enzyme testing, and thermal amplitude studies, may not be readily available in all laboratories, particularly in resource‐limited settings. However, the systematic approach outlined, prioritizing stepwise antibody identification, careful interpretation of reaction phases, and correlation with transfusion history and phenotype—can be adapted to different contexts. In settings with limited resources, targeted use of selected cells, close collaboration with reference laboratories, and reliance on detailed clinical and transfusion histories may help achieve safe and effective transfusion management. Despite these adaptations, access to extended phenotyping and rare donor registries remains important to optimize care for patients with multiple alloantibodies.

## 7. Conclusion

The management of patients with multiple alloantibodies against RBCs requires a complex rule‐based system for blood immunohematology. This necessitates interpreting serologic findings in relation to the phenotype, history of transfusions, and the characteristics of the antibody in the patient. To ensure safe transfusions, immunohematology plays a significant role in the identification and classification of RBC antibodies. Effective management needs excellent testing facilities and skilled personnel. These preventive measures, such as antigen matching, studies on previous antibodies of the patients, immunohematology tests, and typing of the blood groups of the patients in respect to ABO are essential to avoid alloimmunization.

## Author Contributions

Conceptualization and supervision: Haya Wasel and Ahmed Alharbi. Methodology: Abdulmohsen Alotaibi, Jalal Hassan, Saleh Almutairi, Rayyan Alotaibi, and Hiyam Almutairi. Data collection/curation: Maram Alonayzan, Majd Al Anazi, Hajer Aziz, and Atheer Aloqayli. Formal analysis: Eman Almutairi. Writing – original draft: Ahmed Alharbi. Writing (review & editing): Eman Almutairi and Nourah AlHarethi. Resources: Bandar Al Qahtani, Sanad Al Harthi, and Ahmed Shareefi. Funding: None. Visualization: Sarah Al Otaibi, Sarah Abdullah Alobaidi, and Rawan Alfraidi.

## Funding

No external funding was obtained in this research.

## Ethics Statement

Written informed consent was obtained from the patient for publication of this single‐case report and accompanying images. All patient data have been fully de‐identified to ensure confidentiality, with removal of all direct identifiers. This report complies with applicable data protection and privacy standards. According to institutional policy, ethical approval was waived for this single‐patient case report.

## Conflicts of Interest

The authors declare no conflicts of interest.

## Data Availability

The data that support the findings of this study are available upon request from the corresponding author. The data are not publicly available due to privacy or ethical restrictions.

## References

[1] Yazdanbakhsh K. , Ware R. E. , and Noizat-Pirenne F. , Red Blood Cell Alloimmunization in Sickle Cell Disease: Pathophysiology, Risk Factors, and Transfusion Management, Blood. (2012) 120, no. 3, 528–537, 10.1182/blood-2011-11-327361. 22563085 PMC3401213

[2] Dinardo C. L. and Bonini-Domingos C. R. , Alloimmunization in Patients With Sickle Cell Disease: Risk Factors and Impact on Morbidity, Revista Brasileira de Hematologia e Hemoterapia. (2014) 36, no. 3, 202–206.25031060

[3] Schonewille H. , Van de Watering L. M. G. , and Brand A. , Red Blood Cell Alloantibodies After Transfusion: Factors Influencing Incidence and Specificity, Transfusion. (2006) 46, no. 2, 250–256, 10.1111/j.1537-2995.2006.00708.x. 16441603

[4] Harmening D. M. , Modern Blood Banking and Transfusion Practices, 2019, 6th edition, F. A. Davis.

[5] Fung M. K. , Eder A. F. , Spitalnik S. L. , and Westhoff C. M. , Technical Manual, 2020, 20th edition, AABB Press.

[6] Klein H. G. and Anstee D. J. , Mollison’s Blood Transfusion in Clinical Medicine, 2014, 12th edition, Wiley-Blackwell.

[7] Smith D. , Aye T. , Er L. S. , Nester T. , and Delaney M. , Acute Hemolytic Transfusion Reaction due to Anti-P1: A Case Report and Review of Institutional Experience, Transfusion Medicine and Hemotherapy. (2019) 46, no. 5, 380–383, 10.1159/000490897. 31832064 PMC6876609

[8] Henningsen K. , On the Heredity of Blood Factor P, Acta Pathologica Microbiologica Scandinavica. (1949) 26, no. 5, 769–785, 10.1111/j.1699-0463.1949.tb00781.x. 15404595

[9] Holodick N. E. , Rodríguez-Zhurbenko N. , and Hernández A. M. , Defining Natural Antibodies, Frontiers in Immunology. (2017) 8, 10.3389/fimmu.2017.00872, 872. 28798747 PMC5526850

[10] Thapa S. , Jagannathan L. , Mathur A. , Reddy T. V. , and Chakraborty S. , Systematic Approach in Identification and Management of Multiple Alloantibodies: A Case of Triple Alloantibody, Global Journal of Transfusion Medicine. (2019) 4, no. 1, 93–95, 10.4103/GJTM.GJTM_41_18.

[11] Sachan D. , Tiwari A. K. , and Dara R. , et al.Patient Blood Management in a Patient With Multiple Red Cell Antibodies Undergoing Liver Transplant in South India: A Team Approach, Asian Journal of Transfusion Science. (2020) 14, no. 1, 74–78, 10.4103/ajts.AJTS_54_18. 33162713 PMC7607982

[12] Fluit C. R. , Kunst V. A. , and Drenthe-Schonk A. M. , Incidence of Red Cell Alloantibodies After Multiple Blood Transfusions, Transfusion. (1990) 30, no. 6, 532–535, 10.1046/j.1537-2995.1990.30690333485.x. 2378025

[13] Ontario Regional Blood Coordinating Network. (n.d.) , Transfusion Technical Resource Manual, 2015, https://transfusionontario.org/en/resources/.

[14] Roback J. D. , Grossman B. J. , Harris T. , and Hillyer C. D. , Technical Manual, 2011, 17th edition, AABB Press.

[15] Flegel W. A. , Pathogenesis and Mechanisms of Alloimmunization, Transfusion Medicine and Hemotherapy. (2015) 77, no. 4, 191–194, 10.1159/000438845.

[16] Srivastava R. and Agarwal A. , Rare Blood Groups and Rare Donor Program: Indian Perspective, Asian Journal of Transfusion Science. (2012) 6, no. 2, 109–110, 10.4103/0973-6247.98927.

[17] Kaur R. and Jain A. , Rare Blood Donor Program in the Country: Right Time to Start, Asian Journal of Transfusion Science. (2012) 6, no. 1, 1–2, 10.4103/0973-6247.95041. 22623833 PMC3353620

[18] Vichinsky E. P. , Current Issues With Blood Transfusions in Sickle Cell Disease, American Journal of Hematology. (2001) 68, no. 4, 287–291, 10.1002/ajh.10006. 11206956

[19] Yazer M. H. and Triulzi D. J. , Detection of Red Cell Antibodies in a Prospective Cohort of Hospitalized Patients, Transfusion. (2007) 47, no. 10, 1851–1857, 10.1111/j.1537-2995.2007.01407.x. 17880611

[20] Vassallo R. R. , Cold-Reactive Antibodies: Clinical Significance and Laboratory Methods, Immunohematology. (2012) 28, no. 4, 157–163.

[21] Poole J. and Daniels G. , Blood Group Antibodies and Their Significance in Transfusion Medicine, Transfusion Medicine Reviews. (2007) 21, no. 1, 58–71, 10.1016/j.tmrv.2006.08.003. 17174221

[22] Issitt P. D. and Anstee D. J. , Applied Blood Group Serology, 1998, 4th edition, Montgomery Scientific Publications.

[23] Daniels G. , Human Blood Groups, 2013, 3rd edition, Wiley-Blackwell.

[24] Westhoff C. M. , The Rh Blood Group System in Review: A New Face for the Rh Antigen, Transfusion. (2004) 44, no. 11, 1663–1673, 10.1111/j.0041-1132.2004.04237.x. 15504174

[25] Tormey C. A. and Stack G. , The Persistence and Evanescence of Red Blood Cell Alloantibodies in Men, Transfusion. (2009) 49, no. 3, 505–512, 10.1111/j.1537-2995.2008.02014.x. 19040411

[26] Ness P. M. and Shirey R. S. , Alloimmunization in Chronically Transfused Patients: A Dilemma, Transfusion. (1993) 33, no. 10, 857–859.

[27] Daniels G. , Poole J. , de Silva M. , Callaghan T. , MacLennan S. , and Smith N. , The Clinical Significance of Blood Group Antibodies, Transfusion Medicine. (2003) 13, no. 1, 1–22, 10.1046/j.1365-3148.2003.00419.x. 12383334

[28] Evers D. , Middelburg R. A. , de Haas M. , and van der Bom J. G. , Red Blood Cell Alloimmunization in Transfusion-Dependent Patients: A Systematic Review, Transfusion. (2016) 56, no. 11, 2986–2994, 10.1111/trf.13822.27667497

[29] Noizat-Pirenne F. , Tournamille C. , Bierling P. , Le Pennec P. Y. , and Rouger P. , Alloimmunization After Blood Transfusion: Risk Factors and Management, Transfusion Clinique et Biologique. (1998) 5, no. 1, 49–53.

[30] Storry J. R. , Castilho L. , Daniels G. , Flegel W. A. , Garratty G. , and Moldenhauer S. , International Society of Blood Transfusion Working Party on Red Cell Immunogenetics and Blood Group Terminology: Report, Vox Sanguinis. (2014) 106, no. 2, 123–124.

